# Color QR Codes for Smartphone-Based Analysis of Free Chlorine in Drinking Water

**DOI:** 10.3390/s25113251

**Published:** 2025-05-22

**Authors:** María González-Gómez, Ismael Benito-Altamirano, Hanna Lizarzaburu-Aguilar, David Martínez-Carpena, Joan Daniel Prades, Cristian Fàbrega

**Affiliations:** 1 Department of Electronic and Biomedical Engineering, Universitat de Barcelona, Martí i Franquès 1, 08028 Barcelona, Spain; 2 eHealth Center, Faculty of Computer Science, Multimedia and Telecommunications, Universitat Oberta de Catalunya, Rambla del Poblenou, 156, Sant Martí, 08018 Barcelona, Spain; 3 Department of Mathematics and Computer Science, Universitat de Barcelona, Gran Via de les Corts Catalanes, 585, 08007 Barcelona, Spain; 4 Institute of Semiconductor Technology (IHT), Technische Universität Braunschweig, Hans-Sommer Straße 66, 38106 Braunschweig, Germany; 5 Laboratory for Emerging Nanometrology (LENA), Technische Universität Braunschweig, Langer Kamp 6a/b, 38106 Braunschweig, Germany

**Keywords:** free chlorine, QR Code, colorimetric analysis, computer vision, machine learning, water quality

## Abstract

Free chlorine (FC) plays a crucial role in ensuring the safety of drinking water by effectively inactivating pathogenic microorganisms. However, traditional methods for measuring FC levels often require specialized equipment and laboratory settings, limiting their accessibility and practicality for on-site or point-of-use monitoring. QR Codes are powerful machine-readable patterns that are used worldwide to encode information (i.e., URLs or IDs), but their computer vision features allow QR Codes to act as carriers of other features for several applications. Often, this capability is used for aesthetics, e.g., embedding a logo in the QR Code. In this work, we propose using our technique to build back-compatible Color QR Codes, which can embed dozens of colorimetric references, to assist in the color correction to readout sensors. Specifically, we target two well-known products in the HORECA (hotel/restaurant/café) sector that qualitatively measure chlorine levels in samples of water. The two targeted methods were a BTB strip and a DPD powder. First, the BTB strip was a pH-based indicator distributed by Sensafe^®^, which uses the well-known bromothymol blue as a base-reactive indicator; second, the DPD powder was a colorimetric test distributed by Hach^®^, which employs diethyl-p-phenylenediamine (DPD) to produce a pink coloration in the presence of free chlorine. Custom Color QR Codes were created for both color palettes and exposed to several illumination conditions, captured with three different mobile devices and tested over different water samples. Results indicate that both methods could be correctly digitized in real-world conditions with our technology, rendering a 88.10% accuracy for the BTB strip measurement, and 84.62% for the DPD powder one.

## 1. Introduction

Ensuring food safety remains a critical global challenge, particularly in the context of water quality monitoring and sanitation. The European Union has established one of the most comprehensive food safety systems worldwide, featuring regulations and guidelines designed to shield consumers from potential hazards [1]. Despite these substantial endeavors, significant challenges remain in ensuring food safety across the complex supply chain. A primary issue lies in the deficiency of efficient verification and traceability systems, which hinders the monitoring and enforcement of compliance with safety standards. Furthermore, numerous food safety control methods rely on human interpretation, which is susceptible to errors and inconsistencies [2].

In response to this, the digitization of self-monitoring processes has emerged as a promising strategy to bolster the reliability and robustness of food safety plans. By harnessing the capabilities of technology, it becomes feasible to develop tools capable of objectively measuring and recording data, ensuring traceability while minimizing human error. Smartphone-based methods are becoming increasingly popular in the field of self-monitoring for food safety, as mobile devices are now equipped with digital cameras that can serve as optical readers for colorimetric sensors [3,4]. However, the practical deployment of such technologies to read out colorimetric sensors often still depends on the commercialization of additional hardware to house the colorimetric sensors. Even the most compact optical readers occupy additional space and cannot easily be embedded into products [5,6].

To overcome the limitations of current approaches, we propose a smartphone-based solution that combines image analysis with custom-designed Color QR Codes to digitize and interpret free chlorine (FC) tests, as QR Codes are easy to read machine-readable patterns [7], that can be recovered in adversarial scenarios [8,9,10]. This method is embedded in a user-friendly mobile application tailored to support routine self-monitoring in food safety workflows. Our system focuses on two widely used FC detection methods in drinking water: a test strip based on bromothymol blue (BTB), which offers a simple and rapid visual indication of chlorine concentration, and a cuvette-based assay using N,N-diethyl-p-phenylenediamine (DPD), a reference technique in water analysis used due to its high sensitivity and regulatory acceptance [11]. By adapting both tests to a digital format, we enable fast, accurate, and traceable chlorine measurements without the need for specialized instrumentation.

For both digitization pipelines we used a similar approach. At the core of both methods is the use of custom-designed Color QR Codes, which serve as both geometric references and color constancy charts [7]. Additionally, color correction was performed using the embedded references in a Color QR Code, as a simple white balance correction was insufficient for accurate colorimetric readout. To address this, we applied thin plate spline transformations. This approach outperforms simple white-balancing, which is often insufficient for accurately measuring the response of colorimetric dyes [12,13]. Finally, a machine learning framework was used to fit the response data from the colorimetric space to the FC expected response [14].

The performance of both systems was rigorously evaluated under various conditions, including different lighting, mobile devices, and image capture settings. The results demonstrate the potential of these systems to revolutionize water quality monitoring by providing accessible and user-friendly tools for rapid and accurate FC determination.

## 2. Material and Methods

### 2.1. Free Chlorine Sample Preparation and Reagent Methods

Free chlorine (FC) solutions ranging from 0.1 to 8.4 ppm were prepared using a stepwise dilution of sodium hypochlorite (NaClO, 37% *w*/*w*). A 100 ppm stock solution was first obtained by adding 0.57 mL of sodium hypochlorite to 1 L of distilled water. From this stock, a 10 ppm intermediate solution was prepared by diluting 10 mL of the 100 ppm solution into 100 mL of distilled water. Subsequent dilutions were performed in 200 mL volumetric flasks to obtain the desired final concentrations. All solutions were thoroughly mixed and stored in opaque containers to minimize photodegradation.

Concentrations in the low range (0–2.2 ppm) were verified using a DR300 Pocket Colorimeter (Hach Company, Loveland, CO, USA). The Hach DR300 colorimeter used has a reported LOD of 0.02 ppm, as specified by the manufacturer. Measurements below this threshold were not considered reliable and were excluded from quantitative analysis. For samples exceeding the instrument’s upper limit (2.2 ppm), dilution was carried out prior to measurement, ensuring all readings remained within the calibrated range of the device. The associated uncertainty for direct measurements was ±0.02 ppm, and for diluted samples, error propagation was applied according to standard dilution procedures to obtain the final concentration value.

Regarding commercially available reagents to digitize, we targeted two different reagent methods that evaluated different ways to prepare samples and obtain a FC measurement:

- **BTB strip:** We used the Free Chlorine Water Check test from Sensafe^®^ (Industrial Test Systems, Inc., Rock Hill, SC, USA), an assay approved by United States Environmental Protection Agency. This method is designed for testing 50 mL water samples. The procedure involves immersing a single test strip into the 50 mL water sample for 20 s while maintaining continuous back-and-forth motion. The immersion time is adjusted based on the sample temperature, as indicated in the manufacturer’s datasheet. After removal, the strip is gently shaken to eliminate excess liquid, followed by a 20-s wait period before evaluating the color change. The strip employs bromothymol blue (BTB) as a pH-sensitive dye, which exhibits a blue coloration in the presence of free chlorine.
- **DPD powder:** We employed a mixture of reagents commercially known as Permachem Reagents, which contain diethyl-p-phenylenediamine (DPD) dye (Hach^®^, Loveland, CO, USA). The reagent is supplied in single-dose powder packets designed to be mixed with 10 mL of the water sample. The liquid sample was held in custom-designed cuvettes specifically fabricated for this study. In the presence of free chlorine, the DPD reagent reacts to produce a pink coloration, enabling colorimetric quantification.

**Figure 1:**
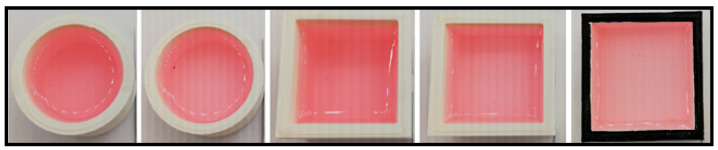
From left to right: different cuvette prototypes for the DPD powder method and the final cuvette design, with internal dimensions of 11.0 mm height, 33.0 mm length, 3.3 mm width. The edges were painted black to improve cuvette detection.

The DPD powder method required the use of a cuvette to hold liquid samples for colorimetric analysis. It is important to note that the perceived color of a translucent liquid depends not only on the depth of the liquid but also on how light is confined within the cuvette. To minimize color distortion during image acquisition, a custom cuvette was designed and fabricated in our laboratory. Several prototypes with varying shapes and internal dimensions were created using 3D printing, employing white polylactic acid (PLA) filament. The prototypes were printed using an Ultimaker 5 3D printer (Ultimaker, Utrecht, The Netherlands) (see Figure 1). The final design (far right in Figure 1), was selected based on its superior performance in minimizing color distortion, plus squared shapes were easy to find using computer vision algorithms, similar to QR Code ones. It features internal dimensions of 1.1 cm (height) × 3.3 cm (length) × 3.3 mm (width), yielding an approximate volume of 10 mL. Finally, once the size of the cuvette was refined in terms of light reflection and color acquisition, the edges of the final cuvette where painted black, which improved the accuracy of automatic cuvette detection within the image processing pipeline.

### 2.2. Design and Fabrication of Color QR Codes

We created two back-compatible Color QR Codes for the digitization of each free chlorine test (BTB strips and DPD powder); these QR Codes acted as colorimetric charts to color correct the scenes of the acquired images. The QR codes shared encoding properties, such as:

- **Data:** A URL to the Diesmar website (https://diesmar.com) along with a unique digital identifier that changes with each new QR code generated, i.e., “n87RXv6i3” (http://diesmar.com/#n87RXv6i3, accessed on 18 May 2025).
- **Modules:** version 3 of the QR Code standard was used, resulting in a 29 × 29 module matrix, which could properly store the data and the colorimetric references.
- **Size:** The physical size of the QR Codes was approximately 1 in^2^ (2.54 cm × 2.54 cm).

**Figure 2:**
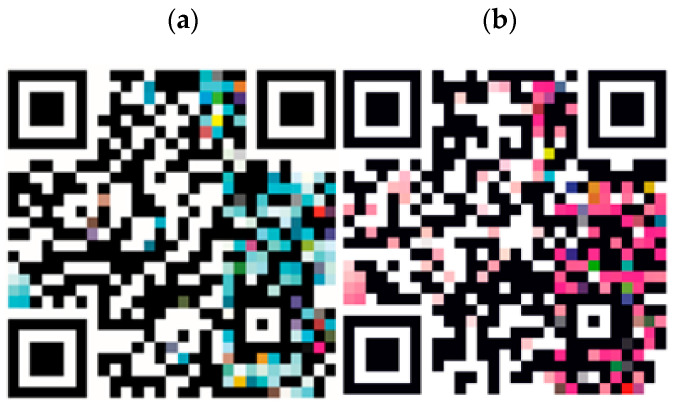
(**a**) QR codes designed for use with Sensafe^®^ chlorine test strips and (**b**) the DPD method (right). The QR codes encode a URL to the Diesmar website and contain a color palette for color correction. The specific URLs encoded are: (**a**) http://diesmar.com/#AdDk (accessed on 18 May 2025) and (**b**) http://diesmar.com/#n87RXv6i3 (accessed on 18 May 2025).

The primary difference between the QR codes is in their respective color palettes:

- **BTB Strip QR Code:** The color palette was created in the CMYK color space and was based on the suggested palette by Sensafe^®^ brand for the product calibration at different chlorine concentrations (see Figure 2a).
- **DPD Powder QR Code:** The color palette was created in the RGB color space and is based on the range of colors exhibited by the DPD reagent when reacting with different chlorine concentrations in our laboratory. To create such a palette, a set of concentration within the range (0.1–10 ppm) were tested and captured with 6500 K lighting and captured with Huawei P20 smartphone model (see Figure 2b).

The back-compatible Color QR Codes were developed using standard Python 3 (>3.9) libraries, with the widely used python-qrcode library serving as the foundation. The codes were printed via commercial offset printing on white Sappi Magno Matt 300 g/m^2^ paper with a polypropylene laminate (Sappi Limited, Johannesburg, South Africa). To ensure accurate color reproduction of the embedded color references, ICC FOGRA27 color profiling was applied during the printing process.

### 2.3. Experimental Setup and Data Acquisition

The series of water samples with known free chlorine concentrations—validated using a Hach colorimeter—were analyzed using both the DPD powder and BTB strip methods. Each sample was imaged within a custom-designed colorimetric setup that incorporated controlled variations in lighting and a standardized reference pattern. To ensure the robustness of the colorimetric models and the reliability of the results, several key factors were carefully considered during dataset construction. In particular, the impact of changing illumination conditions on image capture and color extraction was critically assessed to better emulate real-world scenarios. Furthermore, multiple mobile devices with varying camera specifications were employed to introduce hardware diversity and evaluate the generalizability of the proposed models. As a result, data acquisition was carried out under two distinct conditions: a Fixed Setup, featuring controlled lighting, and a Room Setup, representing standard ambient lighting and more realistic use-case scenarios.

**Figure 3:**
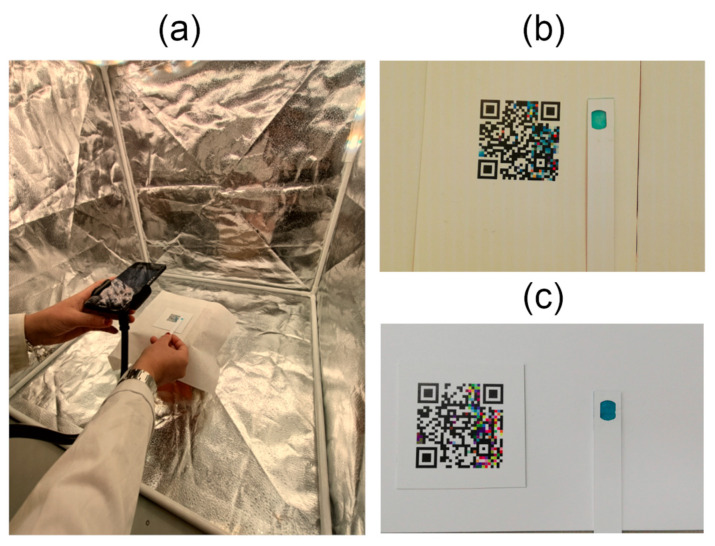
Setup versus image examples from datasets. (**a**) Fixed Setup configured to 2500 K color temperature; (**b**) an image of the BTP strip sensor under the Fixed Setup at 2500 K; and, (**c**) an image of the same sensor under the Room Setup, with laboratory light conditions at, approximately, 4500 K color temperature.

In the Fixed Setup, samples were placed inside a Konseen Professional Photo Light Box (Shaoxing Shangyu Meisen Photography Co., Ltd., Shaoxing, China). To minimize shadows, LED strips were placed around the top of the box to provide uniform illumination from all directions. Variable light conditions were achieved using Phillips Hue Light^®^ LED strip (Signify N.V., Eindhoven, The Netherlands); see Figure 3. Light temperatures ranged from 2500 K to 6500 K in 500 K increments. For image acquisition, a Huawei P20 smartphone (featuring 20 megapixels (MP) Monochrome (f/1.6) + 12 MP RGB (f/1.8)) was mounted on a tripod to ensure consistent framing and focus. In the Room Setup, ambient lighting from the laboratory environment was used to simulate real-world conditions. Images were captured using four different smartphones: Huawei P20, iPhone SE (12 MP, (f/1.8)), Xiaomi Mi A2 (12 MP, (f/1.75) + 20 MP), and Motorola Moto G6 (12 MP (f/1.8) + 5 MP (f/2.2)). To assess the influence of additional lighting, each sample was photographed both with and without flash. Images were taken without fixed positioning, replicating user variability in typical real-life applications.

When available the Open Camera application was used across smartphones, in Android devices. For iPhone the native OS camera application was used; QR Codes were read out as a part of the post-processing pipeline using OpenCV [15]. Subsequent image processing, color analysis, and statistical modeling were carried out using mainly a Python distribution, with several Python packages such as the PyData NumPy stack [16], OpenCV [15] and scikit-learn [14]. Table 1 and Table 2 summarize all the variables for the Fixed Setup and the Room Setup experiments, respectively.

### 2.4. Pattern Recognition and Color Correction

The QR Code was used to locate the specific Region of Interest (ROI) in the captured scenes. Locating the barcode helped us to locate the subsequent hardware component, e.g., the strip or the cuvette, for both methods. Later, for each hardware, a method was implemented to obtain the measurement of color. Then color was corrected using the color references embedded in the QR Code. Next, we detail the pipeline in five steps:

1. **Image acquisition:** An image of the QR Code placed adjacent to the reacted test strip or cuvette was captured using the smartphone camera.
2. **QR Code detection:** The QR Code was located by computer vision algorithms to retrieve barcodes from different surfaces [8], then decoded, and the encoded URL and digital identifier were retrieved.
3. **Color correction:** As the ID of the QR Code is known, our algorithm identified the predefined locations of the color patches within the QR Code. Then, it proceeded to register the colors against saved values of those colors (the same colors under the Fixed Setup with 6500 K light and the Huawei P20 smartphone). These pairs of colors, captured and reference, were used to construct an advanced color correction map, based on non-liner color maps using thin-plate splines [12,13].
4. **ROI extraction:** For each of the specific hardware a ROI was extracted,
5. BTB strip: a feature matcher, the Haar cascade scheme from OpenCV, was used to retrieve features like the strip ink window.
6. DPD powder: a custom feature extractor was used based on a contour detection, plus some simple aspect ratio relations of the square depicted by the cuvette.
7. **Color measurement of the ROI:** The average color value was measured from the captured image. Then, for each image the recovered color correction map was applied [13,17]. RGB measured values were converted to HLS (hue, light, saturation) to feed the different colorimetric models with both the RGB and HSL triplets.

### 2.5. Colorimetric Model

Following the pattern recognition and color correction process, the extracted color features were utilized to develop predictive models capable of estimating chlorine concentration levels in water. Two independent models were developed for each analytical method: a classification and a regression task. The classification task aimed to resolve whether the free chlorine concentration was in a certain low range specific for each test. The second task, regression, aimed to recover a measurement of the free chlorine concentration in reference to the tagged values from the Hach device.

For the classification task, a support vector machine (SVM) classifier [14] was trained using different colorimetric features for each sensor within their respective dataset. The classification was a 3-class classification task as samples were relabeled as: “low concentration”, “acceptable concentration” and “excess chlorine”. These classes meant something different in each of the datasets; for the BTB strip test:

- Low concentration: ≤0.2 ppm
- Acceptable range: 0.2–0.9 ppm
- Excess chlorine: ≥0.9 ppm

and, for the DPD powder test:

- Low concentration: ≤0.2 ppm
- Acceptable range: 0.2–1.1 ppm
- Excess chlorine: ≥1.1 ppm

following their respective manufacturers’ indications.

For the regression task, the relation between the RGB and HSL features and the free chlorine concentration was assessed, for both sensor tests. For the BTB strip test, one of the colorimetric features was directly used for this. For the DPD powder, a PCA was applied to reduce dimensionality and extract the component capturing the most variance in the feature space. This was then used as a univariate predictor in the regression model.

For both tasks, a train–test split of 80–20% was performed for both datasets; the test–train splits remained the same for each of the tasks within each dataset.

## 3. Results and Discussion

### 3.1. BTP Strip Method

**Figure 4:**
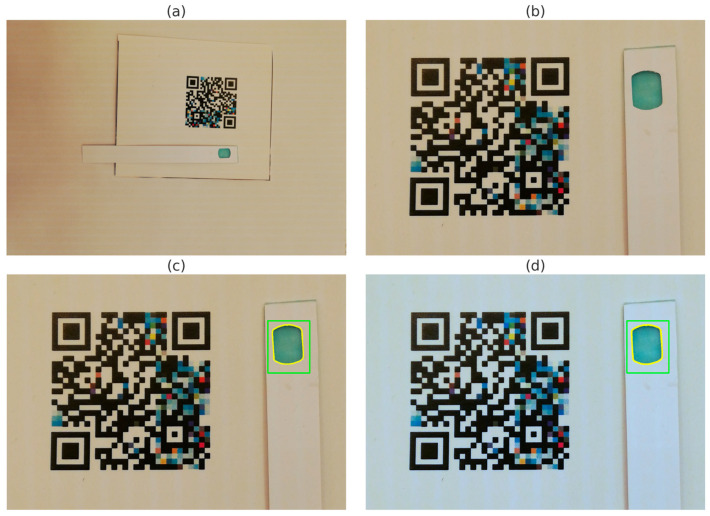
Image processing pipeline for BTP Strip test detection and ROI identification. The figure contains: (**a**) the original image showing the QR Code and test strip, with the QR Code outlined in red; (**b**) the cropped region with the localized QR Code and test strip, again with the QR Code in red; (**c**) ROI identification on the test pad, with the green contour indicating the bounding box from Haar cascade matching and the yellow contour outlining the actual detected shape; and, (**d**) the same ROI as in (**c**), corrected to a standard 6500 K illuminant to enhance color fidelity.

A first qualitative result was obtained regarding the computer vision pipeline to capture the samples of the BTP strip test. Our object segmentation pipeline was based upon QR Code detection [8] and training a well-known Haar-cascade scheme worked precisely [15,18]. First, we detected the Color QR Code (see Figure 4a); then, we corrected the image to an intermediate image where the strip was to be expected at the right side of the barcode (see Figure 4b) Subsequently, the matching-pattern algorithm identified the location of the test strip within the image (see Figure 4c) Finally, the image was color-corrected following the previously mentioned spline fitting methods [12]; see Figure 4d. This pipeline was successfully applied to all images in the dataset without any noticeable issues.

**Figure 5:**
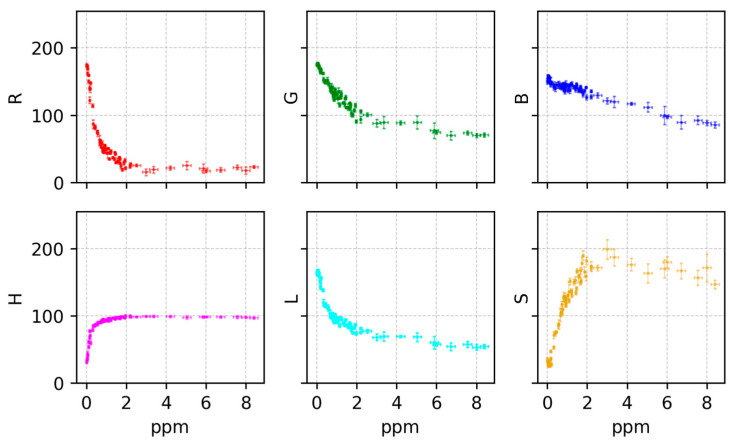
Relationship between the color features of extracted ROI and free chlorine concentration measured with the Hach sensor for the BTP strip commercial method. From left to right, top to bottom: red, green, blue, hue, lightness and saturation. Error bars represent the standard deviation from pixels in the same ROI for each of the measurements.

Once all the images were preprocessed in such fashion, to identify the most informative color features for chlorine determination, six colorimetric features (red (R), green (G), blue (B), hue (H), saturation (S), and lightness (L) [19]) were extracted from images of test strips exposed to varying chlorine concentrations. Figure 5 illustrates the relationship between these color features and the labeled free chlorine concentration. Clear trends were observed between free chlorine concentration and the R, G, and L features, particularly in the 0–4 ppm range. The R component exhibited an approximately exponential decay, while G and L showed more linear decreases as concentration increased. In contrast, H remained relatively constant, and S showed non-monotonic behavior, peaking around 2–3 ppm.

**Figure 6:**
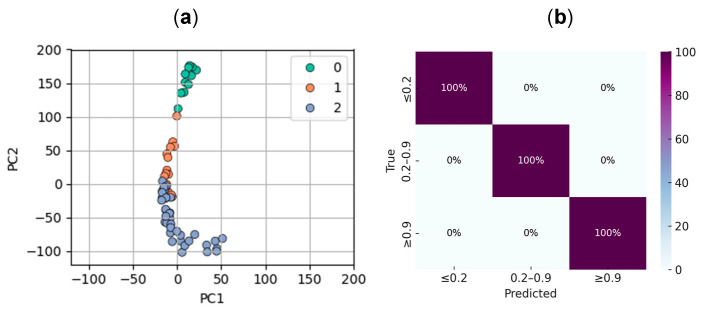
Steps of the classification pipeline. (**a**) Data points after PCA from 6 to 2 components, class 0 represents low concentrations, class 1 represents medium and class 2 represents high concentrations. (**b**) Confusion matrix for validation set, which consisted in data points from the Fixed Setup. Although the dataset is unbalanced (2 samples in class 0, 3 in class 1, and 8 in class 2), the classifier achieved perfect predictions across all classes, as shown by the 100% values on the diagonal.

To classify chlorine concentration into three discrete categories—low (≤0.2 ppm), medium (0.2–0.9 ppm), and high (≥0.9 ppm), as defined by the test strip manufacturer—we trained a support vector machine (SVM) classifier using the six extracted colorimetric features (R, G, B, H, L, and S). Prior to training, the features were normalized to ensure comparability across scales. To visually assess the separability of the data, a principal component analysis (PCA) was performed, reducing the six-dimensional feature space to two components. This transformation preserved over 97.9% of the original variance and revealed a clear clustering structure aligned with the target concentration classes (Figure 6a).

For model development, the dataset was randomly split into training (80%) and validation (20%) subsets, maintaining class balance. Prior to training, the features were auto-scaled using standard normalization (zero mean and unit variance) to ensure comparability across channels and to improve model performance. The resulting SVM model achieved an accuracy of 89% on the training set and 100% on the validation set. The confusion matrix for the validation set (Figure 6b) confirmed perfect agreement between predicted and actual classes, demonstrating the classifier’s capacity to reliably distinguish between the three concentration levels under the controlled conditions of the Fixed Setup dataset.

In parallel, we developed a simple regression model to estimate the chlorine concentration within the medium range (0.2–0.9 ppm). Rather than employing multivariate techniques, we focused on the red channel intensity, which showed the strongest monotonic relationship with concentration in this interval. A univariate model using the natural logarithm of the red channel (ln(R_a_)) yielded high R^2^ values and narrow confidence intervals, offering an interpretable sub-model that complements the classification approach.

**Figure 7:**
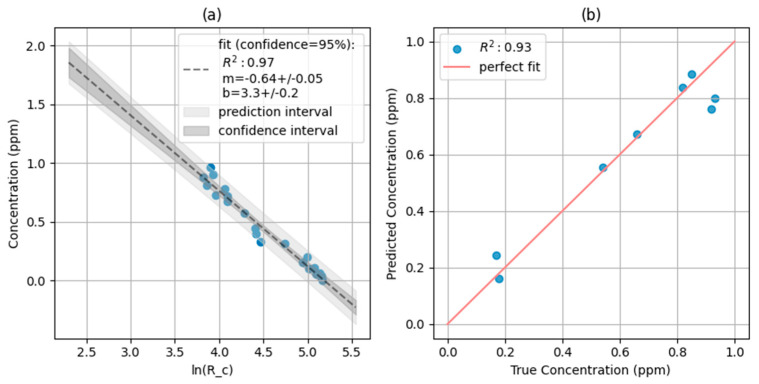
Linear regression model for chlorine concentrations below 0.9 ppm using $\ln{(R}_{c})$ as the predictor. (**a**) Regression model fitted to the training data (dashed line) vs. the validation data (blue dots) with 95% confidence (light gray) and prediction (dark gray) intervals. (**b**) Predicted vs. true chlorine concentration for training (light blue) and validation (blue) datasets. R^2^ values are shown for both training and validation sets. The equation of the fitted line is shown in the top left of the graph.

Additionally, for samples classified within the medium 0.2–0.9 ppm range, a regression task was performed. A linear regression model was introduced to predict the precise free chlorine concentration. Based on the observed relationships in Figure 5, the natural logarithm of the corrected red channel value $\ln{(R}_{c})$ was used as the independent variable. The linear regression model, fitted to the training data, is shown in Figure 7, along with the results on the validation set. The model achieved an R^2^ of 0.97 on the training set and 0.93 on the validation set, indicating a strong linear correlation between $\ln{(R}_{c})$ and the free chlorine concentration. The equation of the fitted line, with a slope of −0.64 ± 0.05 and a y-intercept of 3.3 ± 0.2, can be used to predict chlorine concentration from $\ln{(R}_{c})$ values. The narrow confidence and prediction intervals further demonstrate the model’s precision.

**Figure 8:**
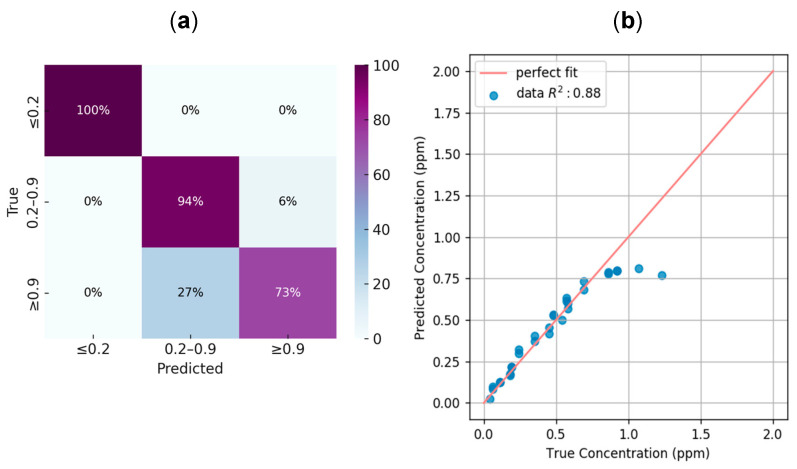
Evaluation of the final model using images captured without flash on a Huawei P20 smartphone. (**a**) Normalized confusion matrix for the classification task across concentration ranges, achieving an overall accuracy of 88.1%. The dataset was moderately unbalanced, containing 9, 18, and 15 samples for the ≤0.2 ppm, 0.2–0.9 ppm, and ≥0.9 ppm classes, respectively. Most misclassifications occurred between medium and high concentrations. (**b**) Regression analysis for the samples classified within the 0.2–0.9 ppm interval, with a coefficient of determination $R^{2}=0.88$, indicating a strong correlation between predicted and true concentrations.

Finally, for the BTB strip method, a test dataset built at room light conditions (Room Setup) was used as test split for a series of smartphones and images were taken for the same labeled chlorine concentrations as the Fixed Setup dataset. For each image, the same image was taken with flash and another one without it. Not all smartphones were used to capture every sample. Images from some devices were excluded from the evaluation dataset, as their lower image quality or inconsistent capture conditions introduced variability that interfered with model performance. Both tasks were tested under this dataset: (1) the classification task that used R, G, B, H, L, S features for the whole three chlorine ranges and (2) the regression task that used only the R features in the medium chlorine range. Figure 8 depicts the detailed metrics of one of the smartphone cameras for both tasks, e.g., a confusion matrix for the classification task and a linear fit against the expected values for the regression task. Figure 8b includes a few misclassified points that lie outside the valid 0.2–0.9 ppm range but were passed to the regression model due to incorrect classification. These points deviate from the linear trend and are shown to highlight the limitations of applying regression to misclassified samples. Table 3 details the results for both tasks for each smartphone and a global accuracy of the classification model. All regression against a perfect fit performed between 80 and 100%, except with Motorola G6, which exhibits the worst results in the dataset.

### 3.2. DPD Powder Method

**Figure 9:**
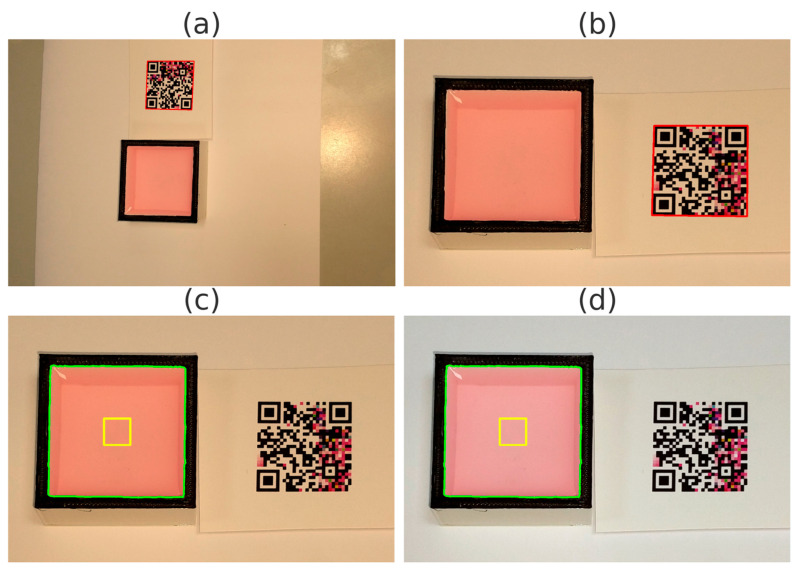
Image processing pipeline for DPD powder test detection and ROI identification. The figure contains: (**a**) the original image showing the QR code and powder cuvette, with the QR code outlined in red; (**b**) the cropped region with the localized QR code and powder cuvette, again with the QR code in red; (**c**) cuvette identification, with the green contour indicating the inner contour and the yellow contour outlining the actual ROI used for measurement; and, (**d**) the same ROI as in (**c**), corrected to a standard 6500 K illuminant to enhance color fidelity.

Similarly to the BTP strip method, we successfully implemented a computer vision pipeline to extract the cuvette of the DPD powder method and measure a ROI region inside the cuvette with the powder colorant. The two main differences fell in the placement and volume of the object to be recognized and the volume of the object itself (bigger than the Color QR Code itself; see Figure 9a,b). For this pipeline, the cuvette was placed on the right side of the machine-readable pattern. This ensured the deformation of the projective transformation between the original image and the cropped image was minimized [8]. Plus, another algorithm was used to recover the color measurement, this was a classical edge-contour detection, using state-of-the-art OpenCV functions [15]. A centered inner ROI approximately 25 times smaller than the cuvette (5 times for x and y axis) was chosen (see Figure 9c,d).

**Figure 10:**
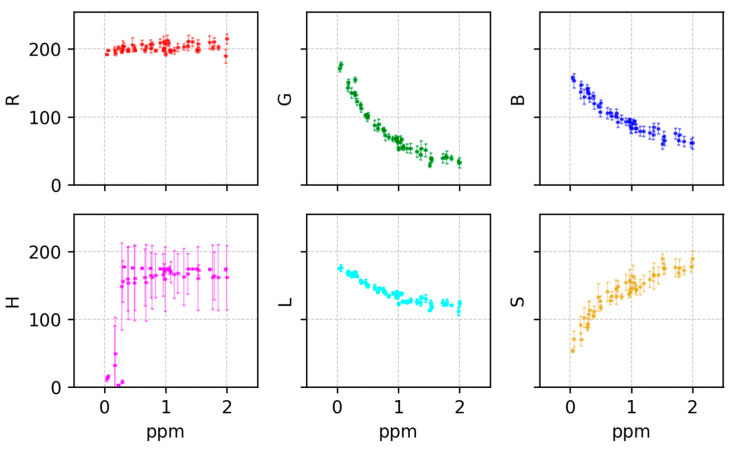
Relationship between the color features of extracted ROI and free chlorine concentration measured with the Hach sensor for the DPD powder test. From left to right, top to bottom: red, green, blue, hue, lightness and saturation. Error bars represent the standard deviation from pixels in the same ROI for each of the measurements.

Again, to determine the most effective color features for quantifying free chlorine concentration, six parameters (red (R), green (G), blue (B), hue (H), saturation (S), and lightness (L)) were extracted from the ROI of images representing various chlorine concentrations. Figure 10 displays the contribution of each component. Despite this, not all the features were used for the DPD powder models. First, as seen in Figure 10, the best contributions to the model were the blue and green channels;, therefore, we used those two components to build similar tasks, as before. For the SVC, both were used without any PCA. The next difference from the BTP strip models is that our boundaries changed due to following another manufacturer specifications, so the boundaries for the classification task were as follow: low was ≤0.2 ppm; medium, 0.2–1.1 ppm; and, high, ≥1.1 ppm. Later, for the medium concentration range, a regression task was trained against the first component of a PCA performed over the logarithm of the G and B features, as opposed to the model for the BTB strip method which used the R feature; this a logical approach as the DPD powder test changes color in the reddish gamut, hence following color theory does not present changes in the red channel of the measurement. We focused on the two most predictive features (G and B channels) and used PCA to construct a univariate representation capturing the dominant variance. This simplified model yielded good accuracy within the target range.

**Figure 11:**
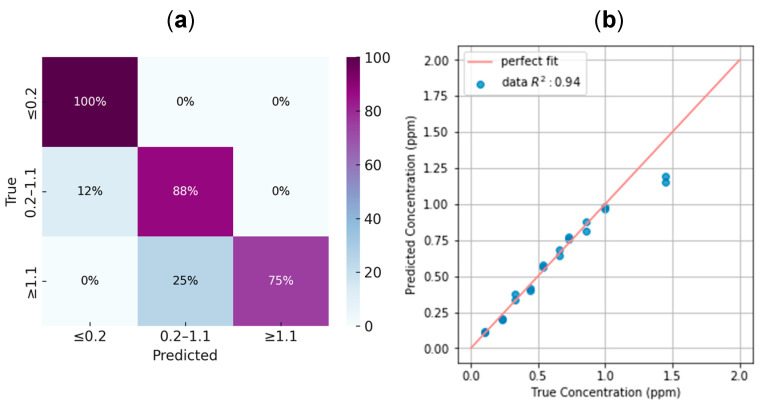
Evaluation of the final model for the DPD powder test using images captured without flash on a Huawei P20 smartphone. (**a**) Normalized confusion matrix for the classification task across concentration ranges, achieving an overall accuracy of 88.1%. The dataset was imbalanced, with 2, 16, and 8 samples corresponding to the ≤0.2 ppm, 0.2–1.1 ppm, and ≥1.1 ppm classes, respectively. Most misclassifications occurred between the medium and high concentration ranges. (**b**) Regression analysis for the samples classified within the 0.2–0.9 ppm interval, with a coefficient of determination $R^{2}=0.88$, indicating a strong correlation between predicted and true concentrations.

As with the BTB strip method, the DPD powder test also yielded strong quantitative results. For instance, using the Huawei P20 smartphone in the Room Setup without flash, the classification task achieved an accuracy of 84.6%, while the regression model reached an R^2^ of 0.94, as shown in Figure 11. The use of flash was avoided for the DPD powder test due to unwanted reflections caused by the cuvette. Table 4 summarizes the results obtained across all smartphones tested under the Room Setup conditions.

### 3.3. Smartphone Application

In parallel with the training of machine learning models and the digitization of the chlorine test samples, we developed a mobile application aimed at demonstrating the potential integration of this technology into a product for prospective clients in the HORECA (hotel/restaurant/café) sector. The resulting application, named *Selfytest*, was designed to assist non-specialist operators in performing fast and reliable free chlorine measurements on-site, using only a smartphone and a printed Color QR Code. The *Selfytest* app managed a local database that stored user information, sample metadata, and measurement results. Additionally, it communicated with a cloud infrastructure via a dedicated REST API, through which the trained colorimetric models were deployed. This architecture ensured platform independence and allowed model updates to be performed server-side without requiring user intervention.

**Figure 12:**
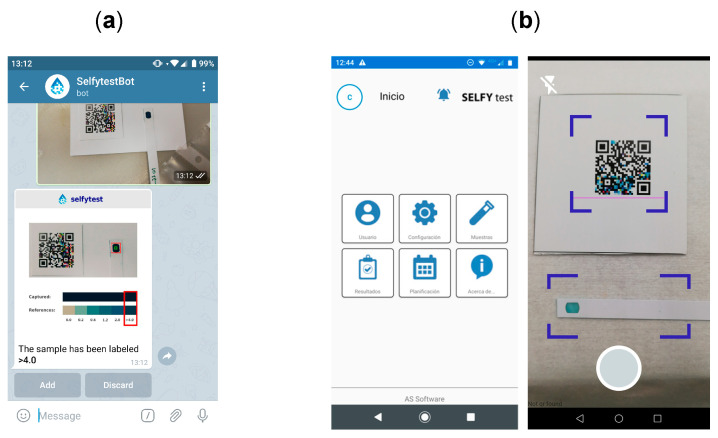
Different stages of the *Selfytest* application. (**a**) Mockup of the *Selfytest* application, as a Telegram bot: a prototype interface was designed to simulate the expected behavior of the final mobile application. It shows automated detection of the test area, comparison with reference color bars, and the corresponding classification of free chlorine concentration. (**b**) Screenshots of the actual *Selfytest* mobile application: the main menu (center) and the image acquisition stage (right), where the QR Code and the test strip are detected in real-time for colorimetric evaluation.

Figure 12 illustrates the main components of the smartphone platform. Figure 12a shows a mockup of the *SelfytestBot*, a prototype messaging interface used in early-stage demonstrations to simulate image acquisition and classification. It displayed automatically extracted result alongside a visual comparison with predefined color references. Figure 12b presents screenshots of the actual *Selfytest* application: the central panel shows the home interface, which allowed users to access configuration options, sample management tools, and result history; the right panel shows the live acquisition screen, where the system detected the QR code and test strip prior to image capture. This system addressed specific needs of the HORECA sector, where decentralized and routine monitoring of water quality was essential, yet often constrained by the lack of laboratory infrastructure or trained personnel [20]. By integrating advanced computer vision and machine learning into a practical mobile interface, the *Selfytest* platform offered a promising, low-cost, and scalable solution for on-site chlorine monitoring.

## 4. Conclusions

In this work, we presented a complete pipeline for smartphone-based digitization of free chlorine (FC) colorimetric assays, combining computer vision techniques with custom-designed Color QR Codes for geometric alignment and color correction. Two commercially available methods—the BTB strip test and the DPD cuvette test—were analyzed using machine learning models tailored for both classification and regression tasks. High performance was achieved across diverse smartphone devices, with classification accuracies exceeding 88% and regression R^2^ values consistently above 0.8. The BTB method showed strong linearity in *ln*(R_a_) response, particularly in the medium concentration range, and benefited from flash illumination due to improved contrast. In contrast, the DPD method achieved its best results without flash, as cuvette reflections under artificial lighting negatively affected color consistency.

In parallel, a prototype mobile application, *Selfytest*, was developed to facilitate practical deployment. The app interfaces with a cloud-based inference engine and includes features for sample tracking and user management, addressing the operational needs of the HORECA sector [20]. Overall, the proposed system demonstrates the feasibility of using smartphones and QR-based references to digitize chemical assays with high reliability.

Future work should focus on revisiting the machine learning pipeline to propose new algorithms, i.e., a mix of both colorimetric sensor data could be studied to develop a sort of “colorimetric nose”, an analogy with the term “electronic nose” for gas sensing devices that use the data from several sensors to measure one or several quantities [21]. Also, the color correction step could be enhanced, and advanced color correction techniques involving deep-learning models could be implemented [22,23]. Moreover, advanced features could be developed for our user-friendly mobile application. Ultimately, these advancements represent significant progress in water quality monitoring, providing rapid, accurate, and accessible methods for determining free chlorine concentration, empowering communities to ensure access to safe drinking water.

## Figures and Tables

**Table 1:** Summary of experimental variables for the Fixed Setup.

Variable	Value	Sample Size
**Commercial test**	BTB strip, DPD powder	2
**Chlorine Concentration (Hach, w/o dilution)**	0 to 2.2 ppm	65
**Chlorine Concentration (Hach, with dilution)**	2.2 to 8.4 ppm	7
**Illumination** **(Hue Lights)**	2500, 3000, 3500, 4000, 4500, 5000, 5500, 6000, 6500 K	9
**Mobile Device**	Huawei P20	1

**Table 2:** Summary of experimental variables. (*) The flash was not used in the DPD experiments. (**) The iPhone SE was not used in the DPD experiments.

Variable	Value	Sample Size
**Commercial test**	BTB strip, DPD powder	2
**Chlorine Concentration (Hach, w/o dilution)**	0 to 2.2 ppm	65
**Chlorine Concentration (Hach, with dilution)**	2.2 to 8.4 ppm	7
**Illumination** **(Room)**	~4500 K	1
Flash *	Yes, No	2
**Mobile Device**	Huawei P20, Xiaomi A2, Motorola G6, iPhone SE **	4

**Table 3:** Evaluation results for the BTP strip method. The model was originally trained using Fixed Setup and tested in the Room Setup. The overall classification accuracy is 91.7%, regression performed R^2^ > 80% for most cases exceptionally for the Motorola G6 and iPhone (with flash).

Smartphone	Flash	Classification (Accuracy %)	Regression (R^2^)
Huawei P20	No	88.1	0.88
Huawei P20	Yes	92.5	0.82
iPhone SE	No	91.3	0.87
iPhone SE	Yes	95.8	0.79
Motorola G6	No	87.0	0.78
Motorola G6	Yes	96.6	0.80
Xiaomi A2	No	100.0	0.96
Xiaomi A2	Yes	94.7	0.79

**Table 4:** Evaluation results for the DPD powder method. The model was originally trained using Fixed Setup and tested in the Room Setup. The overall classification accuracy is 92.7%, regression performed R^2^ > 80% for all smartphones.

Smartphone	Classification (Accuracy %)	Regression (R^2^)
Huawei P20	88.1	0.94
Motorola G6	100.0	0.82
Xiaomi A2	90.0	0.91

## Data Availability

The raw data supporting the conclusions of this article will be made available by the authors on request.
